# Effect of stable and fluctuating temperatures on the life history traits of *Anopheles arabiensis* and *An. quadriannulatus* under conditions of inter- and intra-specific competition

**DOI:** 10.1186/s13071-016-1630-2

**Published:** 2016-06-14

**Authors:** Craig Davies, Maureen Coetzee, Candice L. Lyons

**Affiliations:** Wits Research Institute for Malaria, Faculty of Health Sciences, University of the Witwatersrand, Johannesburg, South Africa; Vector Control Reference Laboratory, Centre for Opportunistic, Tropical and Hospital Infections, National Institute for Communicable Diseases, Sandringham, Johannesburg, South Africa; Present address: Plant Protection Research (PPR), Agricultural Research Council, Vredenburg, Stellenbosch, South Africa

**Keywords:** *Anopheles arabiensis*, *Anopheles quadriannulatus*, Community ecology, Competition, Development rate, Life-history, Survival, Temperature

## Abstract

**Background:**

Constant and fluctuating temperatures influence important life-history parameters of malaria vectors which has implications for community organization and the malaria disease burden. The effects of environmental temperature on the hatch rate, survivorship and development rate of *Anopheles arabiensis* and *An. quadriannulatus* under conditions of inter- and intra-specific competition are studied.

**Methods:**

The eggs and larvae of laboratory established colonies were reared under controlled conditions at one constant (25 °C) and two fluctuating (20–30 °C and 18–35 °C) temperature treatments at a ratio of 1:0 or 1:1 (*An. arabiensis*: *An. quadriannulatus*). Monitoring of hatch rate, development rate and survival was done at three intervals, 6 to 8 h apart depending on developmental stage. Parametric ANOVAs were used where assumptions of equal variances and normality were met, and a Welch ANOVA where equal variance was violated (α = 0.05).

**Results:**

Temperature significantly influenced the measured life-history traits and importantly, this was evident when these species co-occurred. A constant temperature resulted in a higher hatch rate in single species, larval treatments (*P* < 0.05). The treatment 18–35 °C generally reduced survivorship except for *An. arabiensis* in mixed, larval species treatments where it was similar to values reported for 25 °C. Survivorship of both species at 20–30 °C was not significantly impacted and the adult production was high across species treatments. The development rates at 25 °C and 20–30 °C were significantly different between species when reared alone and in mixed species from larvae and from eggs. The effect of temperature was more pronounced at 18–35 °C with *An. arabiensis* developing faster under both competitive scenarios and *An. quadriannulatus* slower, notably when in the presence of its competitor (*P* < 0.05).

**Conclusions:**

The influence of temperature treatment on the development rate and survival from egg/larvae to adult differed across species treatments. Fluctuating temperatures incorporating the extremes influence the key life-history parameters measured here with *An. arabiensis* outcompeting *An. quadriannulatus* under these conditions. The quantification of the response variables measured here improve our knowledge of the link between temperature and species interactions and provide valuable information for modelling of vector population dynamics.

## Background

Increases in average temperatures predicted for the African continent [1] are expected to influence the malaria transmission landscape [2] by affecting key life-history parameters of vector species [3]. Ambient water temperatures can affect a number of traits of the aquatic stages of mosquitoes, in turn influencing adult recruitment [3] and the malaria disease burden. In addition, biological interactions result in nonlinear responses by shifting the competitive landscape to favour one species over another, thereby influencing species abundance and diversity [4]. Across the malaria transmission landscape in Africa, diurnal temperature changes can range between 5 to over 20 °C [5, 6] and these are set to increase in magnitude, notably in maximum temperature extremes in southern Africa [1].

As poikilothermic organisms, the development and survival of mosquitoes is closely tied to the external environment. Temperature conditions influence certain life-history traits, such as survivorship, directly through its impact on larval survival and the resultant adult output as well as indirectly by affecting adult longevity. For instance, temperatures above 30 °C have been shown to decrease adult survival and negatively impact the adult emergence rate by reducing larval survival in *Anopheles gambiae* (*s.s.*) [3]. Several parameters within malaria/mosquito dynamics are influenced to some degree by temperature, including: the probability of infection of the *Anopheles* vector by the *Plasmodium* malaria parasite and therefore, the likelihood of transmission; the rate of infection in the local human population; and equally as importantly, the relative emergence rate of vectors [7]. Defining the temperature-related parameters and quantifying the biological response of malaria vectors, specifically life-history traits, provides valuable information for modelling of vector population dynamics and contributes to our understanding of the factors which determine the malaria disease burden [8].

In north-eastern South Africa, significant distribution overlap exists between the malaria vector *An. arabiensis* Patton and the non-vector *An. quadriannulatus* Theobald, both sibling species of the *An. gambiae* complex, and which are morphologically indistinguishable [9]. Across the African continent however, *An. arabiensis* shows a much broader distribution compared to *An. quadriannulatus*, evident in the absence of the latter species in places such as Madagascar and West Africa [9, 10]. The so-called *An. “quadriannulatus”* in Ethiopia has been shown to be a closely related but distinct species, *An. amharicus* [11], which shares the same polytene chromosomal arrangements and occurs where mean annual rainfall is above 1,000 mm [9]. *Anopheles arabiensis* favours transient pools exposed to sunlight which show significant temperature variation throughout the day [12, 13]. Although observed temperature field data for larvae of *An. quadriannulatus* is sparse, its presence in the same breeding habitats as *An. arabiensis* in the region [14] suggests that as larvae these species may be ecological equivalents, at least in the region where they occur sympatrically. Distribution modelling showing the absence of *An. quadriannulatus* over much of Africa where *An. arabiensis* appears, has been suggested to be under the influence of topography and climate on the former species which likely inhabits areas where the average mean temperature is below the predicted 26.7 °C average for *An. arabiensis*, while the latter appears less affected by these environmental variables [15].

Survival of *An. gambiae* complex members from egg to adult requires a development strategy which takes into account the short lived nature of the breeding site and the ability of the larvae to tolerate temperature extremes. In addition to such abiotic conditions, biotic interactions exist which occur when immatures sharing a breeding habitat come into frequent contact and compete for space and resources [16–18]. The influence that older instar larvae have on con- and hetero-specifics, through cannibalism and predation respectively, significantly reduce survival in sibling species of the *An. gambiae* complex [16, 19]. The presence of a competitor may also have an influence on the age at pupation and *An. arabiensis* larvae have been shown to pupate a day earlier than *An. gambiae* (*s.s.*) in mixed species treatments suggesting a more rapid development rate at a constant temperature of 27 °C [16]. When immatures are reared under higher temperatures (> 30 °C) the production of *An. arabiensis* adults is greater than *An. gambiae* (*s.s.*), although overall production drops as temperatures approach lethal limits [17]. Under semi-field conditions in Western Kenya, habitat sharing between *An. arabiensis* and *An. gambiae* was detrimental for the former with higher mortality rates and earlier time to pupation [18]. The outcome of these interactions is thus context dependent and changes in variables such as temperature, may shift the competitive landscape in favour of one species over another, thereby influencing species abundance and diversity [4]. Due to differences in the vectoral competency and behaviour of each species, changes in the community composition of vectors will have implications for malaria transmission and the resultant control interventions [20].

Considering the effect of temperature on the development and survivorship of anopheline immatures, most studies have been done at constant temperatures and under ideal laboratory conditions (e.g. [16, 17] but see [21]). Relying on extrapolations from these means may be unrealistic and daily temperature dynamics, in addition to means, influence mosquito biology and parasite transmission to a large degree [6, 21–23]. In addition, few studies have investigated the effect temperature has on species interactions in *Anopheles*. There is currently a paucity of information on the thermal biology of *An. quadriannulatus* and, importantly, what effect its co-occurrence with *An. arabiensis* has on the life history of immature forms of the latter species. The relationship between fluctuating temperatures and *An. arabiensis* larval development and survival in the presence of a competitor also remains unclear, and information on the effects of temperature on the larvae of these two species is valuable in light of competition with the vector species.

This study aimed to determine the outcome of inter- and intra-specific competition on the development rate and survival of *An. arabiensis* and *An. quadriannulatus* larvae by controlling ecological variables (competitive scenario and temperature); and to determine whether community composition in the adult stage is regulated by competition under constant and fluctuating abiotic conditions. Improving our knowledge of species interactions at the larval stage in response to increased temperature variability and temperature extremes contributes to our understanding of the current distributions and relative abundances of *An. arabiensis* and *An. quadriannulatus* in southern Africa.

## Methods

### Mosquito colonies and maintenance

Long-established colonies used in these experiments are housed in the Botha De Meillon Insectary at the Vector Control Reference Laboratory in Johannesburg, South Africa. The *An. quadriannulatus* colony, SANGWE, originated from Zimbabwe and has been reared in the laboratory since 1998, whilst the *An. arabiensis* colony, AMAL, originates from the Kruger National Park, South Africa and was established in 2009. The *An. quadriannulatus* colony used in this study represents the designated species A which occurs south of the Zambezi River in southern Africa, in contrast to *An. quadriannulatus* species B known from the Ethiopian highlands [11]. The insectary is kept at a constant temperature of around 25 °C and relative humidity of approximately 80 % with a photoperiod of 12 h light to 12 h dark and simulated dawn/dusk periods of 30 min. Larval stages of all colonies are reared on a mixture of ground Beeno® dog biscuits and yeast extract, while adults are provided with a 10 % sugar solution.

### Experimental design

Adult cages of each colony were provided with a darkened Petri dish serving as an egg plate and females were allowed to oviposit following two blood meals to ensure successful completion of the gonotrophic cycle [24]. The egg plates were left in the cages for six h to minimise variance in hatching. Eggs and recently hatched and unfed larvae (< 24 h old) of each species were transferred into separate temperature treatments using a pipette with 1 cm cut off the tip. Forty larvae or eggs (comprising single species treatments, or a 1:1 ratio of *An. arabiensis* to *An. quadriannulatus*) were introduced into 500 ml containers with distilled water (25 °C) such that the surface area was 175 cm^2^ to maintain a standard density of larvae. Each temperature treatment was replicated five times, randomly assigned in the incubator and switched around daily.

The eggs/larvae were reared under fluctuating temperature regimes mimicking those most likely to exist under natural conditions [12] and which have been shown to be development thresholds in the laboratory [17, 23]: (1) 20–30 °C and (2) 18–35 °C (Panasonic MIR-154 Cooled Incubator, Gunma, Japan); or at a constant temperature of 25 °C in the insectary. Within the range of temperature treatment (1) the development rate of *An. arabiensis* has previously been shown to be linear becoming non-linear above 32 °C [23] with no development occurring at 15 °C and the upper maximum temperature of (2) at 35 °C. Daily light:dark cycles in the incubator were set on a 12:12 h regime cycling from 12 am to 12 pm as in the insectary. Temperatures were set to peak during the day light hours and lower temperatures set to occur during the dark cycle. Larvae in all treatments received optimal amounts of food according to their instar stage. Monitoring of egg hatch and emergence of adults was done at three intervals, six to eight hours apart depending on developmental stage. Adults were collected and killed using ethyl acetate, and the individuals stored in eppendorf tubes with silica for preservation purposes. The rearing conditions and experimental procedure follows closely that of Lyons et al. [23].

Polymerase Chain Reaction (PCR) was used to distinguish between adults from treatments where there was interspecific competition. DNA from the leg or wing from the preserved adults was isolated following standard PCR protocol outlined in Scott et al. [25] using a Bio-Rad Thermal Cycler C1,000 (Hercules, USA). DNA isolates of *An. arabiensis* (AMAL) and *An. quadriannulatus* (SANGWE) were used as controls in PCR and gel electrophoresis.

### Statistical analysis

Survivorship to the adult stage was measured as the proportion of adults that emerged from the initial number of eggs or larvae used in each treatment. Hatch rate of eggs and development rate analyses were conducted on the time it took for 50 % of the eggs to hatch and 50 % adult emergence, respectively. This approach effectively removed late adult emergence outliers. Rates were calculated as rate (days^-1^) = 1/ (t to 50 % life stage/24). No distinction could be made between the hatch times of the two species when in mixed species treatments as the larvae are morphologically identical.

Shapiro-Wilk’s and Levene’s tests were used to assess assumptions of normality and homogeneity of variances, respectively, in SPSS version 20 (IBM Inc., Chicago, USA). When both assumptions were met, a parametric two-way fixed-effect ANOVA was conducted to test the effect of constant and fluctuating temperatures on egg hatch rate, survivorship and development rate followed by the *post-hoc* Tukey HSD test. A fixed effects ANOVA was chosen above a random effects ANOVA because there were less than 5 levels of each factor being tested [26]. In cases where the assumption of homogeneity of variances was rejected, a Welch test was employed using the Games-Howell post-hoc test for unequal variances [27].

## Results

### Larval treatments

**Fig. 1:**
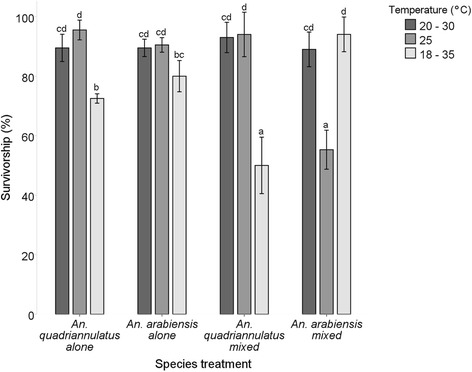
Survivorship (% ± 95 % CI) of first-instars to adults in single species and mixed species treatments of *An. arabiensis* and *An. quadriannulatus* at the two fluctuating temperatures (20–30 °C and 18–35 °C) and a constant temperature of 25 °C. Lower case letters indicate where significant differences in mean survivorship for each group lie (Tukey HSD, *P* < 0.05)

There was a significant effect of temperature (F_(2,48)_ = 35.122, *P* < 0.05) and species (F_(3,48)_ = 6.650, *P* < 0.05) treatment on mean survivorship of first-instar larvae to adulthood (Fig. 1). *Anopheles arabiensis* tolerated higher temperatures better, and *An. quadriannulatus* survival was lower when in the presence of its sibling species at the more extreme temperature treatment. When *An. arabiensis* and *An. quadriannulatus* are combined, interspecific competition resulted in reduced survivorship for each species at 18–35 °C and 25 °C, respectively. Neither species experienced a reduction in survival at 20–30 °C irrespective of the competitive scenario. In single species treatments at 18–35 °C, survivorship was negatively affected.

**Fig. 2:**
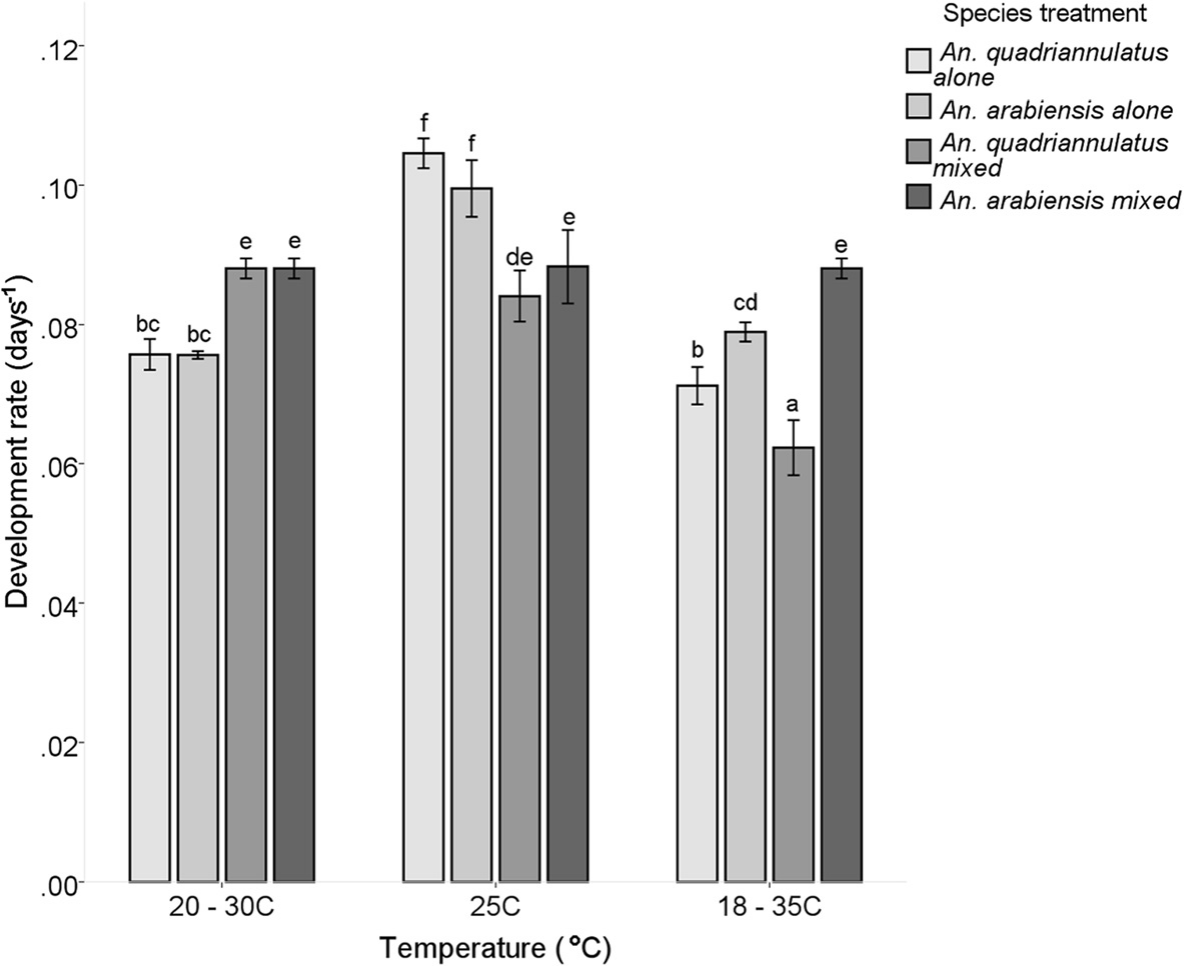
Development rate (days^-1^ ± 95 % CI) of first-instar larvae to 50 % of the adult population in single species and mixed species treatments of *An. arabiensis* and *An. quadriannulatus*, at three temperature treatments (20–30 °C, 25 °C and 18–35 °C). Differences in lower case letters indicate significant differences in mean development rate for each group (Tukey HSD, *P* < 0.05)

The effect of temperature on larval development differed in each competitive scenario. There was a significant effect of temperature (F_(2,48)_ = 181.181, *P* < 0.05) and species (F_(3,48)_ = 25.097, *P* < 0.05) treatment on development rate from larvae to adults. A significant interaction was also observed between the independent variables: temperature and species treatment (F_(6,48)_ = 51.342, *P* < 0.05) (Fig. 2). *Anopheles arabiensis* adults developed faster at the greater fluctuating temperature and emerged sooner (12.67 days in single and 13.45 days in mixed species treatments) compared to *An. quadriannulatus* (14.1 days in single and 13.75 days in mixed species treatments). At 20–30 °C the development rate profiles in single and mixed species were similar, increasing when a species’ heterospecific was present. Across the different temperature treatments, *An. arabiensis* showed the same development response when in mixed species treatments (Fig. 2).

### Egg treatments

When reared alone, the response of hatch rates to temperature treatments were similar between the two species, being fastest at 25 °C (F_(2,36)_ = 16.704, *P* < 0.05) (Tukey HSD, *P* < 0.05). In interspecific treatments there was no significant difference at 25 °C (0.536 days^-1^) in hatch rate compared to 18–35 °C (0.556 days^-1^). The temperature treatment 20–30 °C resulted in the longest time to hatch in all treatments regardless of competitive scenario. In mixed species treatments it was not possible to distinguish between the eggs and first-instars of the two different species, and for this reason, they were lumped together for analysis purposes.

**Fig. 3:**
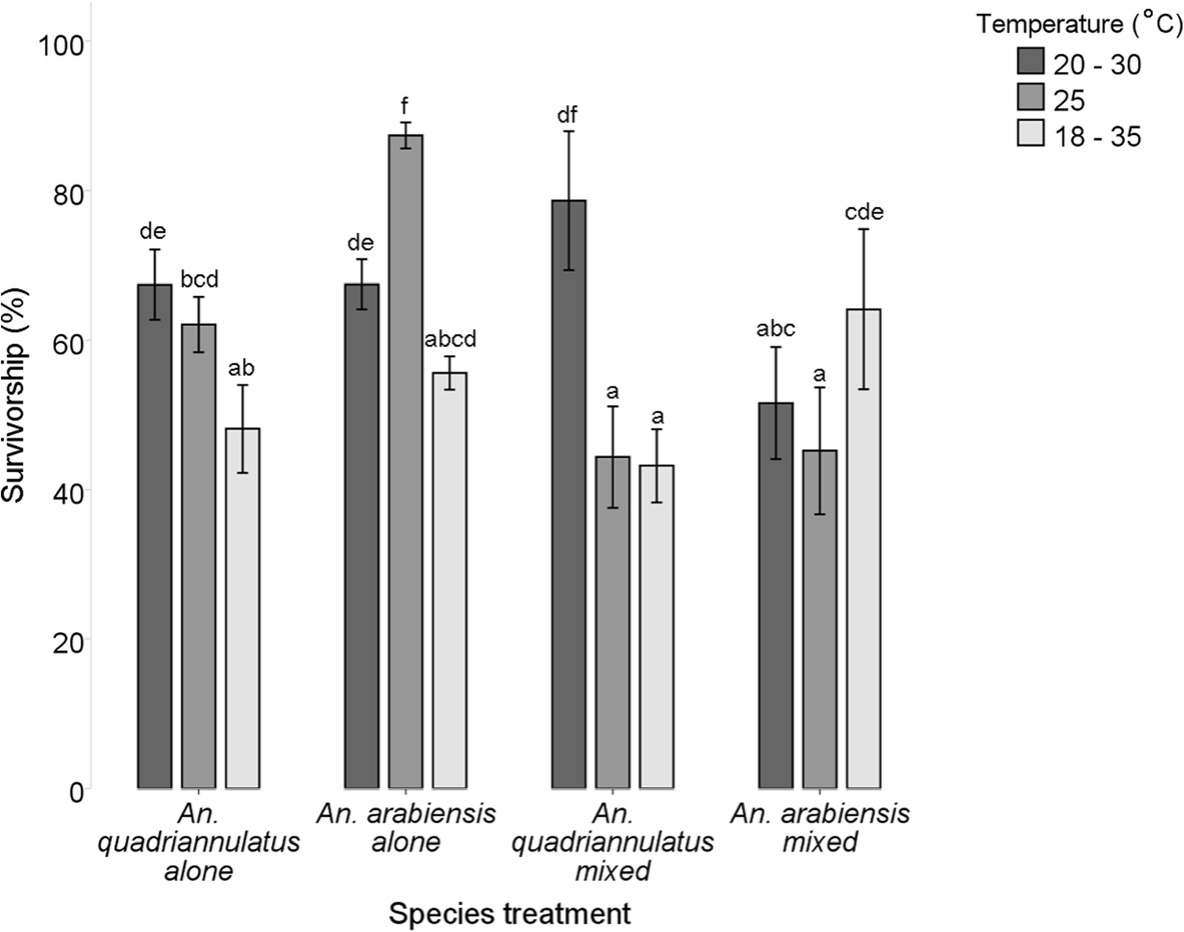
Survivorship (% ± 2SE, 95 % CI) of eggs to adults in single species and mixed species treatments of *An. arabiensis* and *An. quadriannulatus* at the two fluctuating temperatures (20–30 °C and 18–35 °C) and a constant temperature of 25 °C. Differences in lower case letters indicate significant differences in mean survivorship for each group (Tukey HSD, *P* < 0.05)

There was a significant effect of temperature (F_(2,48)_ = 17.940, *P* < 0.05) and species (F_(3,48)_ = 16.199, *P* < 0.05) treatment on mean survivorship from eggs to adults (Fig. 3). A significant interaction was observed between the temperature and species treatment and survivorship (F_(6,48)_ = 22.046, *P* < 0.05). When *An. arabiensis* and *An. quadriannulatus* were reared alone, the number of adults emerging from the initial cohort of eggs was significantly lowered in the temperature treatment 18–35 °C. In interspecific treatments, survival was higher for *An. arabiensis* at 18–35 °C and for *An. quadriannulatus* at 20–30 °C. Survival rates were similar for both species at 25 °C in interspecific treatments when reared from egg to adult (Fig. 3). Survival of *An. arabiensis* in mixed species treatments at 18–35 °C and 20–30 °C was similar.

**Fig. 4:**
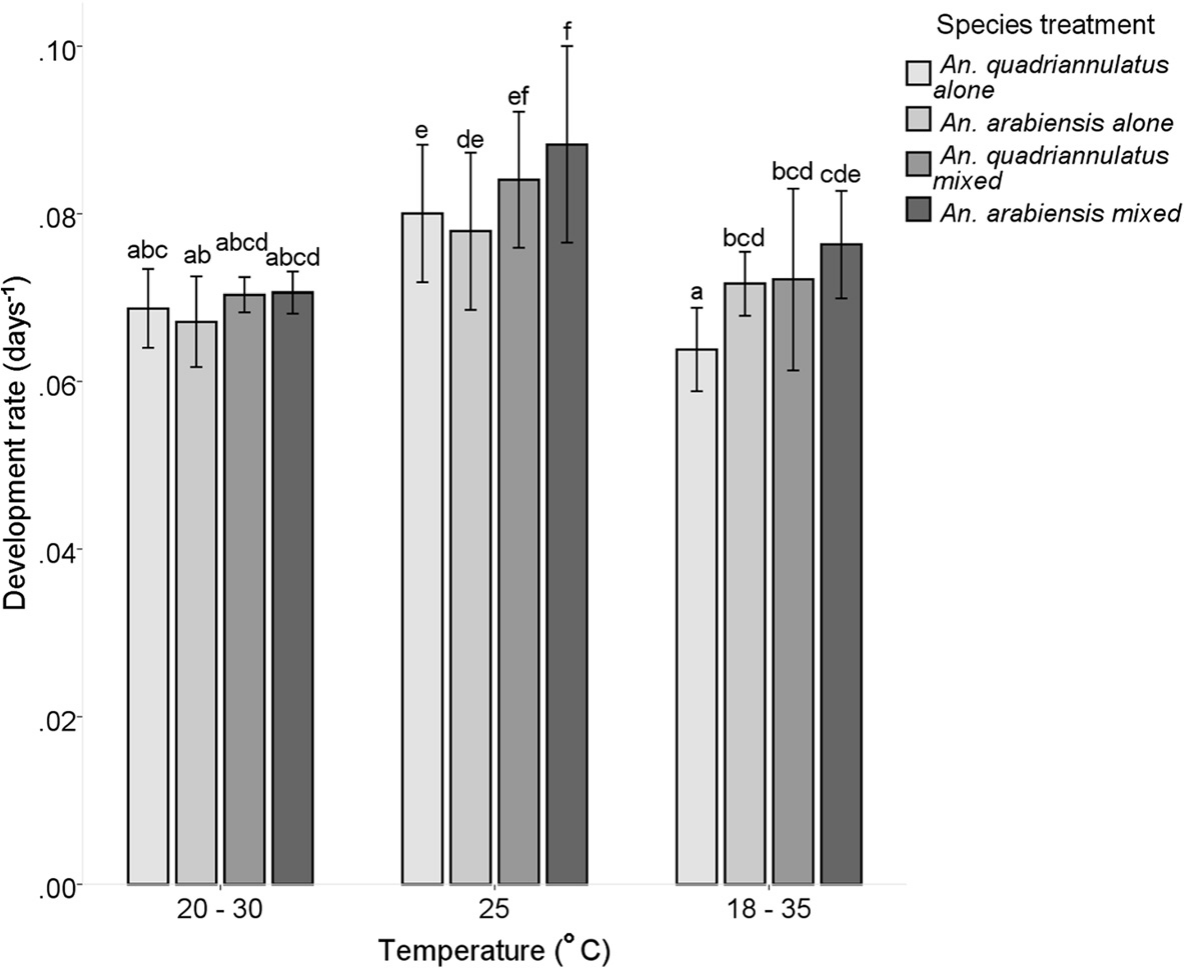
Development rate (days^-1^ ± 2SE, 95 % CI) of the eggs that survived to adulthood (50 % of the adult population) in single species and mixed species treatments of *An. arabiensis* and *An. quadriannulatus* at three temperature treatments (20–30 °C, 25 °C and 18–35 °C). Differences in lower case letters indicate significant differences in mean development rate for each group (Tukey HSD, *P* < 0.05)

The development rate of *An. arabiensis* in mixed species treatments was significantly faster and the adults emerged sooner across all temperature treatments, while for *An. quadriannulatus* it was slower when reared alone at 18–35 °C (Tukey HSD, *P* < 0.05) (Fig. 4). Generally, development rates were slightly slower, regardless of competitive scenario, at 18–35 °C and at 20–30 °C, while the mean development rates of each species were higher at a constant 25 °C (Fig. 4).

## Discussion

Comparing the hatch times, survivorship and development rates of immatures to adults of the vector, *Anopheles arabiensis* (AMAL strain), and non-vector, *An. quadriannulatus* (SANGWE strain), we found different responses to both fluctuating and constant temperatures in single and mixed species treatments. Importantly, these effects were evident when these species co-occurred, with responses in the variables measured being influenced by the presence of a heterospecific. A constant temperature resulted in a higher hatch rate in single species treatments. The treatment 18–35 °C generally reduced survivorship except for *An. arabiensis* in mixed, larval species treatments where it was similar to values reported for 25 °C. Survivorship of both species at 20–30 °C was not significantly impacted and the adult production was high across species treatments. The development rates at 25 °C and 20–30 °C differed between species when reared alone and in mixed species from larvae and from eggs. The effect of temperature was more pronounced at 18–35 °C with *An. arabiensis* developing faster under both competitive scenarios and *An. quadriannulatus* slower, notably when in the presence of its competitor.

Although hatching typically took place within three days, some eggs only hatched after a week. However, the majority of the larvae that hatched did so within the first four to five days, a finding supported in *An. arabiensis* [28] and *An. gambiae* (*s.s.*) [29]. When eggs are exposed to more extreme temperatures for longer periods (relative to the temperature of the rest of the regime), development of the larva inside the egg can be constrained, whereas exposure to brief periods of these same extremes would not be as detrimental [30]. These variations result in staggered hatching amongst egg batches which may be beneficial when the conditions at the breeding site are unpredictable, or when predators/pathogens are present that could be detrimental to the entire egg batch [28, 29].

Survivorship values differed greatly in the current study, with 80–90 % of the first-instar larvae of *An. arabiensis* surviving to adulthood across the temperature treatments and only falling below 60 % when in mixed treatments at 25 °C. Survivorship of *An. arabiensis* was highest when reared in mixed species treatments at 20–30 °C and 18–35 °C. When *An. arabiensis* and *An. gambiae* (*s.s.*) larvae are reared together at a constant 35 °C, the former species dominates, although overall survival is reduced compared to 25 °C [17]. At 25 °C, *An. gambiae* (*s.s*)*.* survival is 20 % higher than that of *An. arabiensis* [16, 17], an observation also observed by Paaijmans et al [18] under semi-field conditions in Kenya. In the current study, the high survival rate of larvae to adults in mixed species treatments of *An. arabiensis* (94 %) compared to *An. quadriannulatus* (50 %) at 18–35 °C suggest the superior competitiveness of the former species over the latter species at more extreme temperatures [17]. This observation reflects our current understanding of at least *An. arabiensis* in Africa which is known to increase in abundance, relative to *An. gambiae* (*s.s.*), during the months of the year with the highest maximum air temperatures [24], and which occurs in regions such as the Ethiopian Rift Valley [31] where water temperatures may reach highs too extreme for *An. gambiae* (*s.s*.) larvae. When reared from eggs, adult survival rates were comparable to those found by Schneider et al. [16] and Kirby & Lindsay [17] in studies on larval competition. At a constant 25 °C, *An. quadriannulatus* is the superior competitor (94 %) over *An. arabiensis* (55 %) in mixed species, larval experiments. In comparison, survivorship was equal when reared at 25 °C from eggs. Lower survival values were also reported in *An. arabiensis* single and mixed species [with *An. gambiae* (*s.s.*)] treatments at this constant temperature [17]. Higher constant temperatures of 30 °C and 35 °C have been shown to lead to reduced survival rates for both species, although survival of *An. arabiensis* was higher than *An. gambiae* (*s.s.*) [17]. At 20–30 °C larval survival rates did not differ markedly across species treatments in the current study. However, survival from egg to adult was higher in *An. quadriannulatus* than *An. arabiensis* at this fluctuating temperature.

Where *An. arabiensis* and *An. quadriannulatus* were reared alone, the significant reduction in survival rates at 18–35 °C may also be attributed to increasing environmental stress on *An. arabiensis* and *An. quadriannulatus*, as the temperatures that the larvae are exposed to for a proportion of the time are not favourable for survival [23]. Temperature variations in the small, often temporary, water bodies in which the larvae of these species exist [12, 13], may show significant temporal variation and are generally not stratified by depth [32]. Exposure to extreme temperatures in species that inhabit temporary, small sites is common. As such, tolerance of higher temperatures compared to anopheline species that occupy larger, more permanent sites, such as *An. funestus*, is important. At this temperature regime, deaths were increasingly common when fourth-instar larvae pupated, as well as in failed eclosion of pupae into adults (pers. obs.), an observation supported by Bayoh & Lindsay [33] in *An. gambiae* (*s.s.*) above 30 °C. One explanation for the failed metamorphosis to pupae and then to adult is that raised temperatures heighten the development rate [23, 33] demanding more rapid uptake of nutrients and a quicker metabolism [34], requirements which may be physiologically demanding, resulting in insufficient mass being accumulated for eclosion [35].

The development rates at 25 °C and 20–30 °C were significantly different between species when reared alone and in mixed species from larvae and eggs. Earlier emergence was reported at 25 °C and at 20–30 °C in single species treatments. No difference in development rate was seen between species in mixed treatments. The effect of temperature was more pronounced at 18–35 °C with *An. arabiensis* developing faster under both competitive scenarios and *An. quadriannulatus* slower, especially when in the presence of its competitor. When reared from eggs, development rates at the two fluctuating temperatures were generally similar. Notable differences in time to eclosion between *An. arabiensis* and *An. gambiae* (*s.s.*) under competition and different water temperatures, with the latter species consistently emerging sooner, have been reported [17, 18]. These findings have been attributed to the larger adult size of *An. arabiensis* which requires greater mass accumulation and thus a longer time spent acquiring resources as immatures [16, 17]. The comparable development rates in the current study suggest the requirements for growth are similar for each species investigated here under the constant 25 °C and the fluctuating 20–30 °C temperature regimes. At 18–35 °C, environmental temperature may have a significant stress effect on the time to adult emergence especially for *An. quadriannulatus* regardless of the presence of its competitor species. The observation in *An. arabiensis* that there is no significant negative effect of fluctuating temperatures incorporating a maximum extreme on development time likely explains the occurrence of this species in drier and warmer conditions, such as northern Botswana, where *An. quadriannulatus* is absent [9].

Habitat sharing by the larvae of these two species would not be detrimental on the development rate of either at moderate temperature conditions, and in fact, may benefit immatures of each as they emerge sooner compared to when reared alone, avoiding the risk that the aquatic habitat will dry up. However, as adults tend to be smaller when emerging sooner [36] their fitness may be diminished and adult longevity reduced [37]. The potential advantages for mosquito larvae to developing faster is an earlier release from threats from competitors, predators and pathogens [38] and potential loss of habitat through habitat flushing, from excessive rainfall [39], or drying up.

Interference competition, leading to insufficient nutrient acquisition, and predation are possible causes of reduced survival and slow development of *An. quadriannulatus* at high temperatures [16, 35]. Interestingly, the more rapid development of *An. arabiensis* at high temperatures did not lead to reduced adult emergence either through failed pupation or eclosion, as has been hypothesised by Chambers & Klowden [35] in *Aedes* and used to explain similar observations in *An. gambiae* (*s.s.*) by Bayoh & Lindsay [33]. At a constant 25 °C, *An. quadriannulatus* appears to exert a predatory effect on *An. arabiensis* survival but not development rate. Predatory behaviour by *An. quadriannulatus* is reported by Koenraadt & Takken [19] as fourth-instars of this species were observed to prey on *An. gambiae* (*s.s.*) first-instar larvae. A mix of different instar stages in a treatment may thus result in predation by one species, which appears to be a facultative process in *An. quadriannulatus,* dependent on temperature.

Improving our understanding of the abiotic parameters influencing malaria vector population dynamics is necessary for modelling malaria transmission. The findings of differential physiological responses to temperature and competitive scenario reported here have implications for understanding the malaria disease burden, especially in the light of high temperature extremes and temperature variability predicted as a result of climate change [1]. However, the limitations of a controlled study need to be considered and semi-field trials will help elucidate better the influence of changing biotic and abiotic variables on the competitive interactions and population dynamics of malaria vectors. It is important to note that these effects are context dependent and that smaller, transient pools may have higher rates of competition compared to larger water bodies where, for instance, predators may have a greater impact on community organisation of adult *Anopheles* species [4]. Moreover, future investigations on larval interactions may wish to determine the influence these conditions have on malaria epidemiology by studying transmission probabilities and other characteristics of anopheline vectors such as adult body size. While such recommendations for future research are admittedly broad, the findings of this study nonetheless give us an insight into the outcome of biotic interactions between closely related species under variable temperatures, important in community ecology terms, and vital for understanding vector-borne disease epidemiology in a given region.

## Conclusions

The current distributions and relative abundances of *An. arabiensis* and *An. quadriannulatus* in southern Africa are unlikely to be influenced by changing species interactions in response to increased temperature variability associated with a warming climate [2]. If high temperature extremes, such as the 18–35 °C investigated here were to become commonplace and extend over a number of weeks, the increased survival and negative effect *An. arabiensis* has on *An. quadriannulatus* where these two occupy the same breeding sites, would favour the former’s survival and could alter the vector borne disease burden as a result of increased adult recruitment. Survivorship of both species at 20–30 °C was not significantly impacted and adult production was high across species treatments. However, the complexities of the effects of changing local conditions, such as temperature, on vector abundance do not only extend to the interactions between these two sympatric species and are an interplay between the influences of the human and ecological settings. Generally, more semi-field experiments are required to improve our understanding of the effects of competition and how this changes under different conditions.

## Abbreviations

ANOVA, analysis of variance; DNA, deoxyribonucleic acid; PCR, polymerase change reaction; Tukey HSD, Tukey (Honest Significant Difference) *post-hoc* test
